# Is there sufficient evidence that the hyoid bone can be used for sexual dimorphism? a systematic review and meta-analysis

**DOI:** 10.1007/s00414-026-03722-3

**Published:** 2026-02-11

**Authors:** Cristina M. Beltran-Aroca, Alba Checa-Poza, Elena De-La-Barrera-Aranda, Eloy Girela-Lopez, Aioze Trujillo-Mederos, Manuel Romero-Saldaña

**Affiliations:** 1 Section of Legal and Forensic Medicine, Faculty of Medicine and Nursing, University of Córdoba, Av. Menéndez Pidal s/n. 14004, Córdoba, Spain; 2 CTS637 Legal and Forensic Medicine. University of Córdoba, Córdoba, Spain; 3 Department of Morphological Sciences and Social Health, University of Córdoba, Córdoba, Spain; 4 GC31 Group, Maimonides Institute of Biomedical Research of Córdoba (IMIBIC), Córdoba, Spain; 5 Department of Physiotherapy, Research and Sport of Córdoba, FISIDEC University Centre (Affiliated with the University of Córdoba), Cabra, Córdoba, Spain; 6 Department of Nursing, Pharmacology and Physiotherapy, Faculty of Medicine and Nursing, University of Córdoba, Córdoba, Spain; 7 GA16 Lifestyles, Innovation and Health Associated Group. Maimonides Institute of Biomedical Research of Córdoba (IMIBIC), Cordoba, Spain

**Keywords:** Hyoid bone, Sexual dimorphism, Forensic anthropology, Sex estimation, Hyoid fusion

## Abstract

**Objective:**

Sex estimation using the hyoid bone is a complementary approach in forensic anthropology, valuable when other skeletal elements are unavailable. Its morphology shows sexual dimorphism and age-related ossification and may assist neck-trauma assessment. However, population variability and methodological heterogeneity limit generalization. This systematic review and meta-analysis evaluate the accuracy and forensic applicability of hyoid-based sex estimation.

**Method:**

Following PRISMA guidelines, a systematic search was conducted in PubMed, Scopus, and Web of Science up to August 31, 2024. Human studies estimating sex from hyoid morphology were included, using macroscopic or imaging analyses with individuals of known sex as reference. Non-human studies, pathologies, and reviews were excluded. Reporting completeness and risk of bias were appraised with STROBE and QUADAS-2. A narrative synthesis and meta-analysis using random-effects models provided pooled sensitivity and specificity estimates.

**Results:**

Of 965 records, 23 studies were included, comprising 4,770 participants. Evidence supported sexual dimorphism of the hyoid, and fusion increased with age. Studies showed moderate-to-high reporting completeness but frequent bias concerns, particularly due to unclear patient-selection and reference-standard domains. The highest accuracies ranged from 69.2% to 93% using discriminant analysis and reached 95.4% with machine-learning approaches. A meta-analysis of six studies (827 participants) yielded pooled sensitivity 0.742 (95% CI 0.514–0.887) and specificity 0.799 (95% CI 0.612–0.909).

**Conclusion:**

The hyoid bone can be useful for sex estimation, especially in limited skeletal preservation. Multivariate and artificial intelligence models show promising performance, but evidence remains limited by heterogeneity. Future studies should standardize procedures and broaden validation across populations.

**Supplementary Information:**

The online version contains supplementary material available at 10.1007/s00414-026-03722-3.

## Introduction

The identification of human remains is a fundamental goal in forensic anthropology and legal medicine. In many scenarios, such as in archaeological contexts [1], mass disasters, cases of advanced decomposition, or when only skeletal remains are recovered, sex estimation becomes a priority, as it informs subsequent steps in the reconstruction of the biological profile [2].

While the pelvis and skull are typically the most reliable sources due to their pronounced sexual dimorphism, these structures are not always preserved or available. The hyoid bone can be assessed both on dry bone and through medical imaging, allowing evaluation of morphology and sexual dimorphism when other skeletal components are unavailable [3, 4]. When preserved, it may offer useful forensic information. In forensic and judicial contexts, accurate sex estimation helps establish biological identity and supports case interpretation. Although the hyoid bone is fragile and less frequently recovered than larger skeletal structures, it has been recognized as a potentially informative element for identification and documentation in medico-legal investigations [5].

Anatomically, the hyoid is a small, U-shaped bone located in the anterior neck between the mandible and the thyroid cartilage. It is unique in that it does not articulate with any other skeletal structure, being suspended in place by a complex arrangement of muscles and ligaments. Its overall shape, which has been classified into symmetrical and asymmetrical types with possible sex-related patterns [6], further contributes to its relevance in anthropological and forensic contexts. Anatomical variation in the hyoid also carries forensic implications. D’Souza et al. [7] reported that thinner greater horns, more frequently observed in females, may help explain the higher fracture susceptibility described in hanging and strangulation cases, reinforcing the relevance of the hyoid in neck-trauma evaluation.

Beyond trauma assessment, several studies have examined sexual dimorphism in the hyoid bone. Males generally present greater overall dimensions, particularly in body width, body height, and cornual length, whereas female bones tend to be smaller and lighter. Wider inter-greater cornu distances in males have also been reported, reflecting broader lateral expansion of the apparatus [8]. In addition, total hyoid weight is generally higher in males, reinforcing the size-related component of dimorphism, and fusion increases with age, with no global association with sex across age groups [5, 9, 10]. These findings support the inclusion of the hyoid in sex estimation protocols, particularly when more commonly used bones are unavailable.

Despite this potential, the reliability of the hyoid for sex estimation is limited by several factors. One is the variability in the fusion of its components, which differs by age and sex. Although fusion status could potentially alter certain morphometric features, Soltani et al. [11] addressed this issue by selecting variables known to be unaffected by age-related fusion, thus enhancing the consistency of their results across different age groups.

Another important factor is population variation: morphometric thresholds may not be universally applicable due to interpopulation differences, highlighting the need for population-specific standards [2, 12]. These limitations underscore the need to explore alternative methodological approaches that may overcome population-specific variability and anatomical constraints.

In this context, while traditional linear measurements remain the most accessible and widely used approach [8], recent studies have explored more sophisticated statistical techniques to improve classification accuracy. In particular, the use of machine learning methods, such as Support Vector Machines or Artificial Neural Networks, has shown promising results, with some models reaching up to 95% accuracy in correctly classifying sex from hyoid morphology [13]. These approaches enable the simultaneous analysis of multiple variables and the optimization of classification criteria. Nevertheless, their application still requires broader validation across diverse populations and forensic contexts [14].

Given these considerations, the present study aims to systematically review the available evidence regarding the forensic utility of the hyoid bone in sex estimation. Specifically, this study aims to evaluate the accuracy of various morphometric and analytical methods, while examining the impact of age and inter-population variation on their performance, and incorporates a meta-analysis of diagnostic accuracy to provide pooled estimates of sensitivity and specificity.

## Materials and methods

### Registration

This systematic review was conducted in accordance with the Preferred Reporting Items for Systematic Reviews and Meta-Analyses (PRISMA) 2020 statement [15, 16]. PRISMA-DTA was not used as the included studies were mainly observational morphometric analyses and only few provided diagnostic accuracy data. The review protocol was registered in the International Prospective Register of Systematic Reviews (PROSPERO) under the registration number CRD42024575403.

## Eligibility criteria

The research question was structured according to the PICOS framework. All included studies were diagnostic or diagnostic accuracy studies. The Population comprised human subjects whose sex was estimated using hyoid bone analysis. The Intervention involved macroscopic or imaging-based examination of the hyoid bone. The Comparison group consisted of individuals of known sex used as a reference. The Outcome of interest was the accuracy or performance of the sex estimation method. Eligible Study designs included observational cross-sectional studies, cohort studies, and case series reporting quantitative results.

Exclusion criteria were applied to studies involving individuals with bone pathology affecting the hyoid, animal models, soft tissue analysis, or combined methods where the contribution of the hyoid was not isolated. Additionally, studies were excluded if they involved individuals of unknown sex, lacked outcome data on sex classification, or were published as reviews, editorials, books, conference abstracts, case reports, or dissertations.

### Information sources

A comprehensive literature search was conducted using PubMed, Scopus and Web of Science. The search covered all literature published up to August 31, 2024, without restrictions on the initial publication date. Only studies published in peer-reviewed journals indexed in these databases were considered, thereby excluding grey literature, and articles had to be written in English or Spanish. The last search was conducted on August 31, 2024.

### Search strategy

The search strategy was developed and executed independently by two reviewers. It combined DeCS/MeSH terms, keywords, and free-text terms using Boolean operators “AND” and “OR” and was adapted to the syntax and filters of each database.

The general search equation included terms related to sexual dimorphism and the hyoid bone, such as: (“sexual dimorphism” OR “sex estimation” OR “sex assessment”) AND (“hyoid bone” OR “laryngohyoid complex”).

For PubMed, filters were applied to the Title and Abstract fields. In Scopus and Web of Science, equivalent adaptations were made using the “Title, Abstract and Keywords” and “Topic” fields, respectively. The full search equation, encompassing all keyword variations and Boolean operators, along with the database-specific search strategies for PubMed, Scopus, and Web of Science, are detailed in the Supplementary Information (Table S1).

### Study selection

**Fig. 1:**
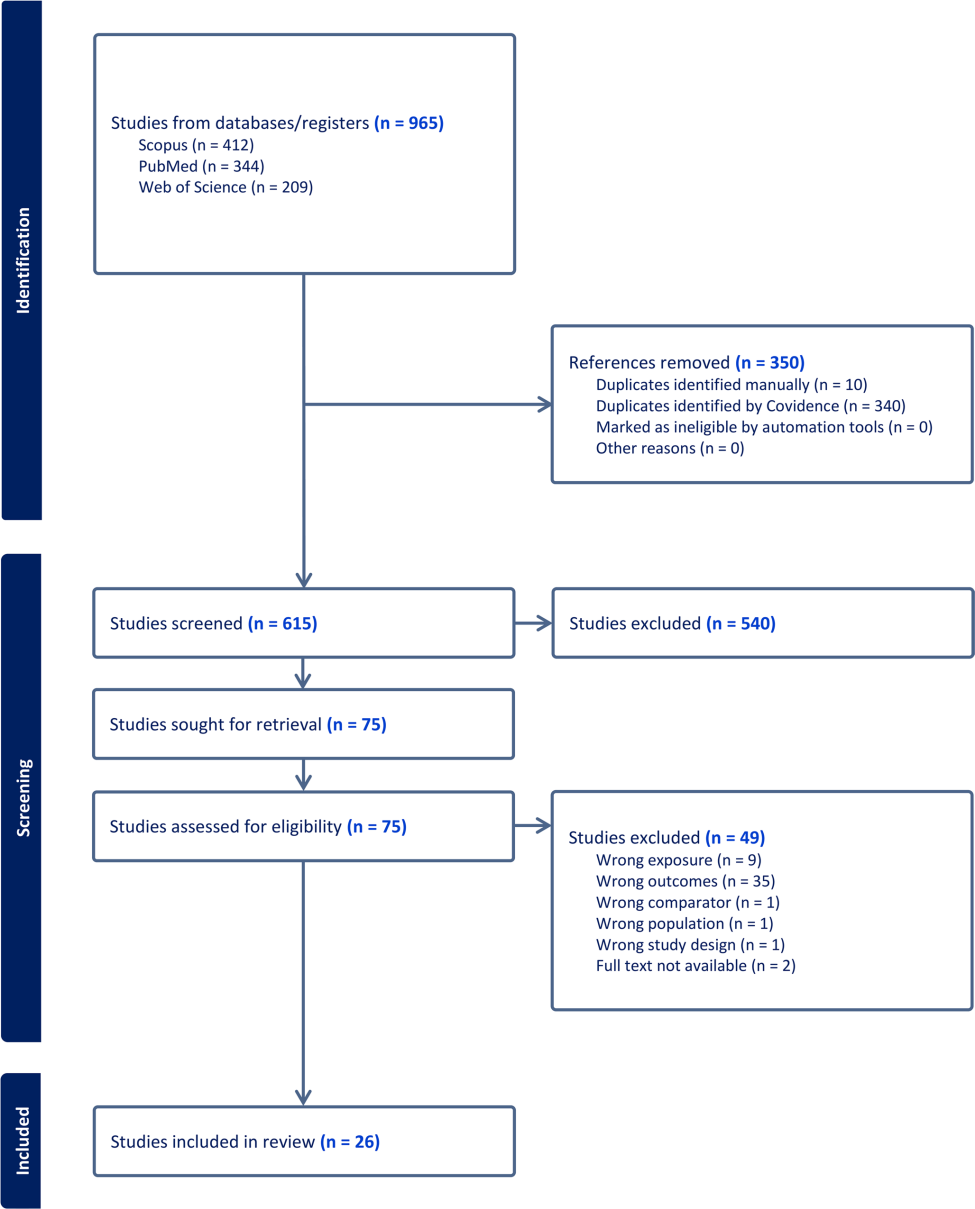
PRISMA flow diagram of the study selection process

All records retrieved from the database searches were exported to Covidence^®^ systematic review software [17], where duplicates were automatically identified and removed. Covidence is a collaborative platform designed to streamline the production of systematic reviews. Two reviewers independently screened the titles and abstracts for relevance. Full texts of potentially eligible studies were then retrieved and assessed against the predefined inclusion and exclusion criteria. Inter-reviewer agreement was moderate at the title and abstract screening stage (κ = 0.76) and substantial at the full-text eligibility stage (κ = 0.88). Discrepancies were initially discussed and resolved by consensus between the reviewers; when consensus could not be reached, a third author was consulted. The study selection process is illustrated in the PRISMA flow diagram (Fig. 1), following the structure recommended in the PRISMA 2020 guidelines.

### Data collection process

Data extraction was performed independently by two reviewers using a structured Excel spreadsheet developed specifically for this review. The spreadsheet was designed to capture relevant information on study design, sample size, population characteristics, variables measured, analytical methods, and key outcomes.

The extracted data were cross-checked for consistency and completeness prior to analysis.

In two cases where data were missing or unclear, the corresponding authors were contacted via email; however, no response was received.

### Data items

The structured Excel spreadsheet used for data extraction included the following items for each study: first author, country, year of publication, study design (prospective or retrospective), sample size, sex distribution (male/female), mean age of participants, biological status (in vivo or ex vivo), data source (dry bone or imaging), fusion status of the hyoid bone (fused or unfused), methods employed, variables measured, statistical analyses performed, percentage of correctly classified individuals, and the authors’ main conclusion.

### Risk of bias in individual studies

Reporting completeness and risk of bias of the included studies were assessed through a combined approach. Given that all studies were observational in nature, the STROBE (Strengthening the Reporting of Observational Studies in Epidemiology) checklist [18] was used exclusively to report how completely key methodological and reporting items were described. This 22-item checklist covers all major sections of observational studies. Although some of the included papers explored the discriminative potential of morphometric features, they were not designed or reported as diagnostic accuracy studies in the strict sense. Their observational nature, and the fact that results were typically presented as morphometric comparisons, regression models, or classification functions rather than structured test-accuracy outcomes, made the STARD or PRISMA-DTA frameworks less applicable. For this reason, STROBE was considered the most appropriate tool to assess reporting completeness in these studies. While STROBE does not assess methodological quality or risk of bias, fuller reporting of key items can indirectly help identify potential bias sources, particularly in domains such as participant selection or outcome reporting. In this sense, its use enhances the transparency recommended by PRISMA 2020. For each article, a reporting completeness index was calculated as the proportion of applicable items adequately reported, expressed as a percentage. Because no universally accepted threshold exists, we pragmatically excluded from the final synthesis studies with reporting completeness below 50%, as they lacked sufficient methodological detail to allow reliable data extraction or meaningful appraisal. Only three of the 26 initially eligible studies were excluded on this basis [8, 19, 20]. For the remaining studies, STROBE-based percentages were used descriptively to summarise reporting completeness and were not interpreted as formal measures of methodological quality or risk of bias. In parallel, the risk of bias was independently assessed by two reviewers using the QUADAS-2 tool for systematic reviews of diagnostic accuracy studies [21, 22]. It evaluates four key domains: patient selection, index test, reference standard, and flow and timing. Each domain was rated as having a low, high, or unclear risk of bias based on a set of signalling questions. Additionally, concerns regarding applicability were considered for the first three domains. Disagreements in STROBE and QUADAS-2 assessments were resolved by discussion, and when necessary, by consultation with a third author, following the same consensus procedure used for study selection. QUADAS-2 judgments were not used as exclusion criteria: all studies were retained in the qualitative synthesis, and the small subset contributing 2 × 2 data was included in the meta-analysis. Risk-of-bias assessments informed the interpretation of both narrative findings and the certainty of the pooled sensitivity and specificity estimates.

### Meta-analysis

A meta-analysis of diagnostic accuracy studies was performed using REVMAN version 5.4 and SPSS version 28. For each included study, the basic diagnostic accuracy measures were calculated: sensitivity (TP/[TP + FN]) and specificity (TN/[TN + FP]). The 95% confidence intervals (CI) for each estimate were obtained using the Clopper–Pearson exact method, which is appropriate for binomial proportions and particularly robust in small samples.

For the meta-analysis of each parameter separately, a classic DerSimonian–Laird (DL) random-effects model was applied on the logit scale, with within-study variances estimated using the delta approximation. The pooled results were transformed back to the probability scale for clinical interpretation. Forest plots of sensitivity and specificity were constructed, showing point estimates, exact CIs for each study, and the pooled effect with its respective CI. The heterogeneity of each meta-analysis was assessed through the following statistics: inconsistency (I2), Cochran’s Q test, and Tau square. According to I², the heterogeneity can be interpreted as not important (0–40%), moderate (30%−60%), significant (50–90%), and considerable (75%−100%).

Additionally, a bivariate random-effects model (HSROC/bivariate model) was fitted for pairs of sensitivity and false-positive rate on a logit scale, allowing simultaneous estimation of the pooled mean and the covariance matrix between studies. The estimation was performed using maximum likelihood. A parametric SROC curve was constructed using the parameterization proposed by Rutter and Gatsonis, along with the diagnostic performance summary point and the ellipse of variability between studies (calculated using the delta method in the probability scale transformation).

## Results

A total of 26 studies met the initial inclusion criteria. However, after assessing reporting completeness using the STROBE checklist, three studies were excluded for scoring below the 50% threshold [8, 19, 20]. Therefore, 23 studies were ultimately included in this systematic review. The selection process is outlined in the PRISMA flow diagram (Fig. 1).

Given the methodological and population heterogeneity among studies, a qualitative synthesis was conducted. The results are presented in three sections:

1. characteristics of the included studies,
2. reporting completeness and risk of bias, and
3. accuracy and statistical findings related to sex estimation based on the hyoid bone. 

In addition, a meta-analysis of diagnostic accuracy was performed on the subset of studies providing suitable data, yielding pooled estimates of sensitivity and specificity.

### Study characteristics

The main methodological and sample characteristics are summarized in Table 1. All studies were published in English and involved the estimation of sex based on hyoid bone morphology. The research was geographically diverse, with most studies conducted in Asia (Turkey, India, Iran) [11, 13, 23–30], followed by Europe (France, Czech Republic, the Netherlands) [3, 31–33], North America [9, 34–36], Africa [37], and Australia [38].

**Table 1:** Methodological and sample characteristics

Author	Year	Country	Design	Sample size	Sex distribution (male/female)	Age range (mean ± SD)	Biological status	Data source	Fusion data reported
Balseven-Odabasi et al. [23]	2013	Turkey	P	85	53/32	21–80	Ex vivo	Digital camera imaging	By sex
Demet Mutlu et al. [13]	2024	Turkey	R	240	120/120	21–90	In vivo	CT imaging	NR
Fakhry et al. [3]	2013	France	R	180	106/74	18–97 (51,37 ± 20,41)	Ex/In vivo	CT imaging	NR
Fisher et al. [9]	2016	USA	R	136	66/70	0–95	In vivo	CT imaging	No sex detail
Guliani et al. [24]	2018	India	P	46	33/13	18–85	Ex vivo	Dry bone analysis	NR
Haj Salem et al. [37]	2020	Tunisia	P	240	123/117	0–81 (36,7 ± 19,6)	Ex vivo	CT imaging	NR
James et al. [39]	2022	India	P	100	50/50	21–70	Ex vivo	Dry bone analysis	Unclear
Kindschuh et al. [34]	2010	USA	R	398	200/198	20–79	Ex vivo	Dry bone analysis	No sex detail
Köse et al. [40]	2022	Turkey	R	130	NR	14–55 (34,37 ± 14,45)	In vivo	CT imaging	Unclear
Logar et al. [35]	2016	USA	R	134	68/66	20–49	Ex vivo	Dry bone analysis	By sex
Miller et al. [36]	1998	USA	P	315	188/127	0–93	Ex vivo	X-ray imaging	No sex detail
Mukhopadhyay [25]	2012	India	P	50	38/12	19–70 (39,72 ± 13,37)	Ex vivo	Dry bone analysis	NR
Naimo et al. [38]	2015	Australia	R	406	210/196	0–100 (35,93 ± 24,15)	Ex vivo	CT imaging	NR
Okasi et al. [26]	2018	Iran	P	372	217/155	13–87	Ex vivo	CT imaging	NR
Pollard et al. [31]	2011	France	P	72	52/20	18–91	Ex vivo	CT imaging	NR
Reesink et al. [33]	1999	Netherlands	P	59	33/26	NR	Ex vivo	X-ray imaging	NR
Soltani et al. [11]	2017	Iran	P	349	173/176	25–54 (39,91 ± 8,13)	Ex vivo	Dry bone analysis	NR
Soltani et al. [27]	2022	Iran	P	248	179/69	18–80 (42,98)	Ex vivo	Dry bone analysis	NR
Torimitsu et al. [41]	2018	Japan	P	280	140/140	18–89	Ex vivo	CT imaging	No sex detail
Tyagi et al. [28]	2019	India	P	286	169/117	14–80	Ex vivo	Dry bone analysis	Unclear
Tyagi et al. [29]	2020	India	P	53	34/19	20–75	Ex vivo	Dry bone analysis	By sex
Tyagi et al. [30]	2021	India	P	293	173/120	15–80	Ex vivo	Dry bone analysis	Unclear
Urbanová et al. [32]	2013	Czech Rep.	P	298	153/145	20–89 (63,58)	Ex vivo	Dry bone and Digital digitizer	No sex detail

NR Not reported, Design: P prospective, R retrospective

Sample sizes ranged from 46 to 406 individuals (4,770 participants in total), with a median of 226 participants. Sex distribution was balanced in approximately half of the studies [11, 13, 34, 39], while others reported a predominance of male subjects [3, 24–26, 28–30]. Only one study included clearly more females than males [9]. Most studies examined adult populations (≥ 18 years), although a few included broader age ranges extending from neonates to elderly individuals [9, 36–38].

Regarding biological status, most studies were based on ex vivo samples [24, 25, 28–30], although three employed in vivo imaging [9, 13, 40]. Approximately half of the datasets were obtained through radiological methods such as CT or X-ray [3, 9, 13, 23, 26, 31, 33, 35–38, 40, 41], while the remainder used dry bone specimens. One study combined both [32].

Fusion status of the hyoid was inconsistently reported. Some studies offered detailed information by sex and fusion type [23, 29, 35], others mentioned the variable without specifics [9, 32, 34, 36, 41] and many did not report it at all.

Most studies followed a prospective design, while approximately one-third were retrospective [3, 9, 13, 34, 35, 38, 40]. All applied statistical procedures to estimate sex based on morphometric measurements of the hyoid bone. As detailed in Table 2, the most frequently used technique was discriminant function analysis, followed by logistic regression [9, 11, 26, 27, 31, 37]. Only one study applied machine learning methods such as support vector machines and artificial neural networks [13]. The accuracy values reported in Table 2 refer to the percentage of correct classification. More than half of the studies achieved classification accuracies above 70%, with one exceeding 95% [13], and only one falling below 50% [24]. Several studies reported sex-specific classification accuracies, with modest to notable differences. Balseven-Odabasi et al. achieved 92.5% accuracy in males versus 78.1% in females [23]. Similarly, Reesink et al. reported 82% for males and 72% for females [33]. In contrast, Kindschuh et al. obtained higher accuracy in females (89.1%) than in males (80.5%) [34]. Demet Mutlu et al., who applied both classical and machine learning techniques, reported nearly identical results for both sexes (approximately 94%) [13]. These findings illustrate that while some studies show sex-related variation in performance, others demonstrate a more balanced classification across sexes. Inter- or intraobserver reliability was addressed in several studies [9, 11, 25, 31, 32, 34, 35, 37, 40].

**Table 2:** Statistical methods and accuracy in sex estimation

Author	Method	Statistical analysis	Accuracy
Balseven-Odabasi et al. [23]	Metric analysis (33 variables)	Descriptive, t-test, DFA	Overall: 78.1% – Male: 92.5% – Female: 78.1%
Demet Mutlu et al. [13]	Metric analysis (8 variables)	Descriptive, DFA, SVM, ANN	DFA: 93.3% SVM: 93.8% – Male: 94.2%, – Female: 92.5% ANN: 95.4% – Male: 95% – Female: 95.8%
Fakhry et al. [3]	Metric analysis + Morphological analysis (12 variables)	Descriptive, t-test, DFA, Pearson correlation, Procrustes coordinates	Overall: – Male: 89.5% – Female: 85.1%
Fisher et al. [9]	Fusion grade analysis + bone density analysis	Descriptive, Chi-square test, ANOVA with Tukey’s post hoc, logistic regression	Bone density: – Overall: 64% – Male: 59% – Female: 69%
Guliani et al. [24]	Metric analysis (6 variables)	Descriptive, linear regression	Overall: 48.4%
Haj Salem et al. [37]	Metric analysis (10 variables)	Descriptive, t-test, logistic regression, ROC curve, DFA	Overall: 73%
James et al. [39]	Metric analysis (7 variables)	Descriptive, t-test, ROC curve, DFA	Overall: 62% – Male: 80% – Female: 44%
Kindschuh et al. [34]	Metric analysis (10 variables)	Descriptive, t-test, ANOVA, DFA	Overall: 82.8–84.4% – Male: 80.5–87.3% – Female: 81.9–89.1%
Köse et al. [40]	Metric analysis (8 variables)	Descriptive, t-test, DFA	Overall: 55.95–81.35% – Male: 22.2–70.4% – Female: 79.5–92.3%
Logar et al. [35]	Metric analysis (15 variables)	Descriptive, t-test, Wilcoxon test, DFA	Fused: – Overall: 93% – Male: 92.6%, – Female: 93.8% Unfused: – Overall: 90.1% – Male: 87.8% – Female: 92%
Miller et al. [36]	Metric analysis (31 variables)	Descriptive, ANOVA, DFA	– Male: 69.2% – Female: 75.2%
Mukhopadhyay [25]	Metric analysis (6 variables)	Descriptive, t-test, DFA	Overall: 90% – Male: 86.8% – Female: 100%
Naimo et al. [38]	Metric analysis (17 variables)	Descriptive, ANOVA with Tukey’s post hoc, linear regression, DFA	Overall: 74.7%
Okasi et al. [26]	Metric analysis (9 variables)	Descriptive, t-test, logistic regression, DFA	Overall: 81.7%
Pollard et al. [31]	Metric analysis (6 variables)	Descriptive, Mann-Whitney U test, ROC analysis, logistic regression	– Male: 32.69–67.31% – Female: 30–70%
Reesink et al. [33]	Metric analysis (13 variables)	Descriptive, t-test, DFA	Overall: 76% – Male: 82% – Female: 72%
Soltani et al. [11]	Metric analysis (3 variables)	Descriptive, t-test, ROC curve, logistic regression	Overall: 72%
Soltani et al. [27]	Metric analysis (4 variables)	Descriptive, t-test, logistic regression	Overall: 67%
Torimitsu et al. [41]	Metric analysis (7 variables)	Descriptive, t-test, DFA	Fused: – Overall: 94.1% – Male: 90.6%, – Female: 98% Unfused: – Overall: 93.3% – Male: 89.7% – Female: 96.7%
Tyagi et al. [28]	Weight analysis (4 variables)	Descriptive, t-test, ANOVA, DFA	Overall: 67.5–76.6% – Male: 62.1–73.4%, – Female: 75.2–81.2%
Tyagi et al. [29]	Weight analysis (3 variables)	Descriptive, t-test, ANOVA, DFA	Overall: 75.5% – Male: 73.5% – Female: 78.9%
Tyagi et al. [30]	Metric analysis (12 variables)	Descriptive, t-test, ANOVA, DFA	Overall: 77.5% – Male: 78% – Female: 76.7%
Urbanová et al. [32]	Metric analysis (9 variables) + Morphological analysis (23 landmarks)	Descriptive, t-test, Hotelling’s T², permutation test, Procrustes (GPA), DFA, symbolic regression	DFA (multivariate): Fused: – Overall: 92.41% – Male: 92.86% – Female: 91.96% Unfused: – Overall: 84.93% – Male: 85% – Female: 84.85% Symbolic regression: Fused: – Overall: 96.34% – Male: 99.06% – Female: 92.94% Unfused: – Overall: 96.43% – Male: 96.43% – Female: 96.43%
*M* male, *F* female, *DFA* discriminant function analysis, *SVM* support vector machine, *ANN* artificial neural network, *ROC* receiver operating characteristic, *GPA* generalized Procrustes analysis, *ANOVA* analysis of variance, *T²* Hotelling’s T-squared test. Where applicable: “Fused” and “unfused” refer to hyoid bone fusion status.			

In addition to methodological aspects and accuracy results, the studies analyzed a wide range of morphometric features of the hyoid bone. The assessed variables were predominantly linear and anatomically grounded. Most analyses focused on the hyoid body (body length, width, and height), the greater and lesser cornua (lengths and intercornual distances), and the spatial relationships between these elements (distances between horns and the body, or between distal ends of the horns). Some studies included additional parameters such as body thickness or concavity depth [11, 13, 32], as well as cross-sectional widths [34, 38]. A small number of studies incorporated segmental weights as morphometric indicators [28, 29]. While most studies relied on fewer than ten variables, several employed complex models with more than fifteen measurements [23, 35, 36, 38]. A complete overview of the morphometric variables assessed, and the authors’ main conclusions is provided in the Supplementary Information (Table S2).

### Reporting completeness assessment (STROBE)

**Fig. 2:**
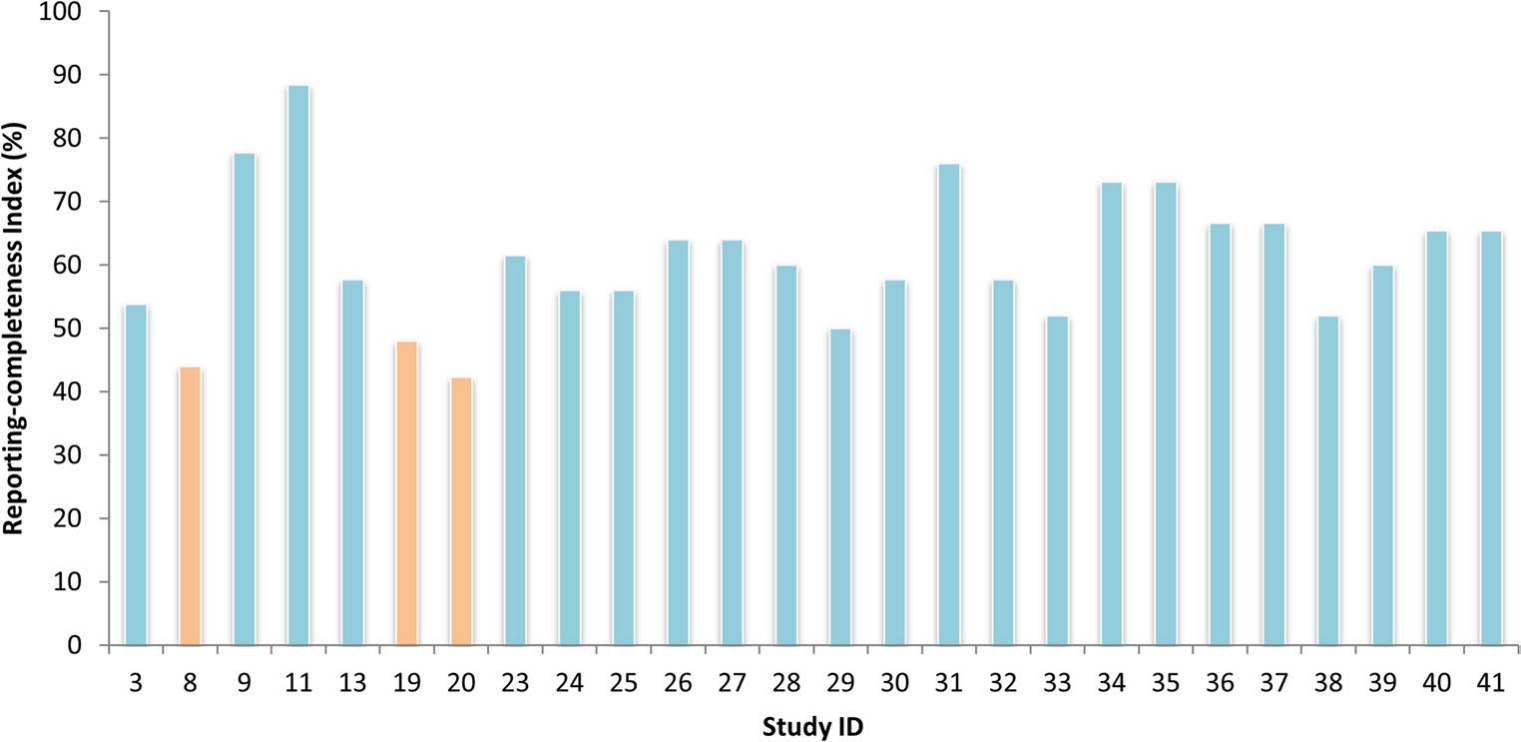
Reporting-completeness index (%) per study (26 studies)

The reporting completeness of the 26 studies initially considered for inclusion was assessed using the STROBE checklist for cross-sectional studies. This tool comprises 22 items rated as “yes,” “no,” or “not applicable,” covering essential aspects of study design, conduct, and reporting. For each study, a final reporting-completeness index was calculated as the proportion of applicable items fulfilled. Three studies scored below the 50% threshold and were therefore excluded [8, 19, 20]. The remaining 23 studies were included in the final synthesis. A summary of the item-level evaluation is provided in the Supplementary Information (Table S3), and a graphical overview of the reporting-completeness distribution is shown in Fig. 2.

More than half of the included studies (*n* = 14) reached an index of at least 60%, with the highest reporting-completeness score [11] exceeding 88%. Certain items were consistently well reported across all studies, particularly title and abstract (item 1b), background/rationale (item 2), data sources/measurement (item 8), quantitative variables (item 11), statistical methods (subgroups and interactions) (item 12b), participants (numbers at each stage) (item 13a), outcome data (item 15), and key results (item 18). In contrast, some aspects were less frequently addressed. For instance, participants (eligibility criteria and methods of selection) (item 6) were not described in three studies [33, 36, 38], and interpretation (item 20) was missing in five cases, with no synthesis of results considering study objectives, limitations, or related literature. Additionally, setting (item 5) was insufficiently reported in one study [32], lacking details on locations and relevant dates.

### Risk of bias assessment

**Fig. 3:**
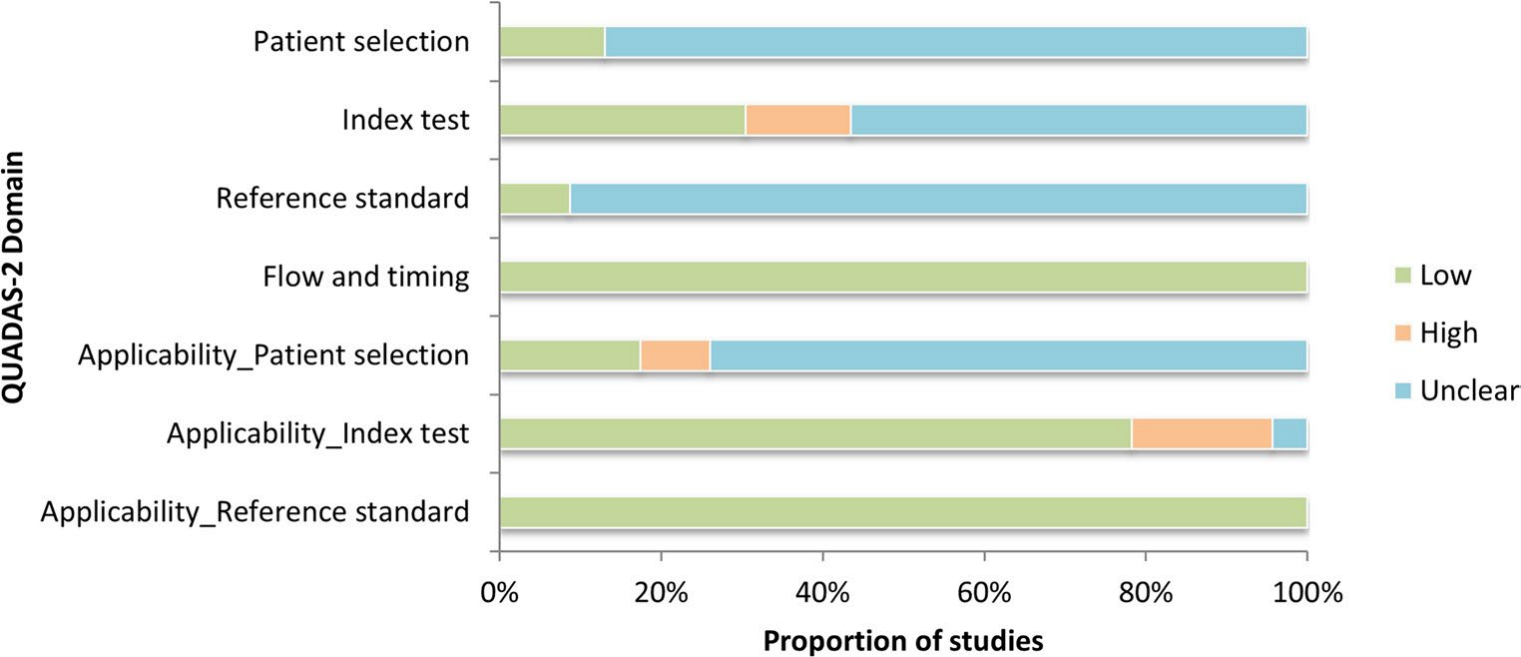
Risk of bias and applicability concerns by domain (QUADAS-2)

Risk of bias was evaluated using the QUADAS-2 tool. The distribution of bias levels and concerns regarding applicability across the included studies is summarized in Fig. 3, while individual domain-level assessments are detailed in Table 3.

**Table 3.**
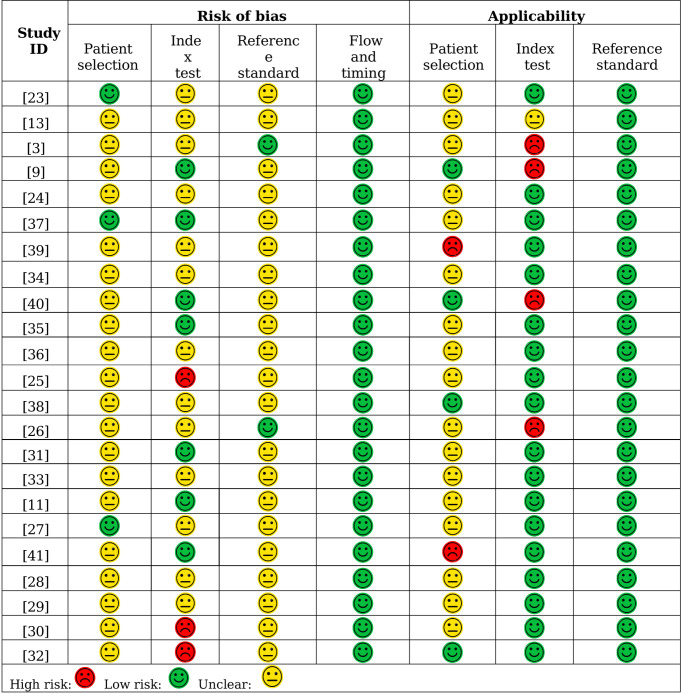
Domain-level risk of bias and applicability concerns

Across the risk-of-bias domains, most studies provided insufficient methodological detail. Patient selection showed the highest proportion of unclear risk (86.96%), with only 13.04% of studies judged at low risk. Similarly, the reference standard domain displayed very limited information, with 91.30% rated unclear and only 8.70% rated low risk. The index test domain exhibited substantial uncertainty, with 30.43% of studies at low risk and most classified as unclear (56.52%) or high risk due to missing information on blinding and decision thresholds. This limits the confidence with which these results can be evaluated. In contrast, flow and timing was consistently well reported: all studies were rated at low risk.

Regarding applicability concerns, patient selection again showed substantial uncertainty (73.91% unclear), reflecting poor reporting of sampling strategies. Index-test applicability was low risk in 78.26% of studies but high in 17.39%. Reference-standard applicability showed the most consistent performance, with all studies rated at low concern.

None of the studies achieved a fully low-risk profile across all QUADAS-2 domains. Nonetheless, one study [37] showed low risk in most domains but still lacked sufficient information in the reference-standard domain, where the only issue was imprecise reporting. This study also achieved a reporting-completeness score close to 70% in the STROBE-based assessment (Supplementary Information Table S3).

Overall, the predominance of unclear judgments, particularly in patient selection and reference standard, indicates methodological underreporting rather than demonstrably low risk.

### Meta-analysis

**Fig. 4:**
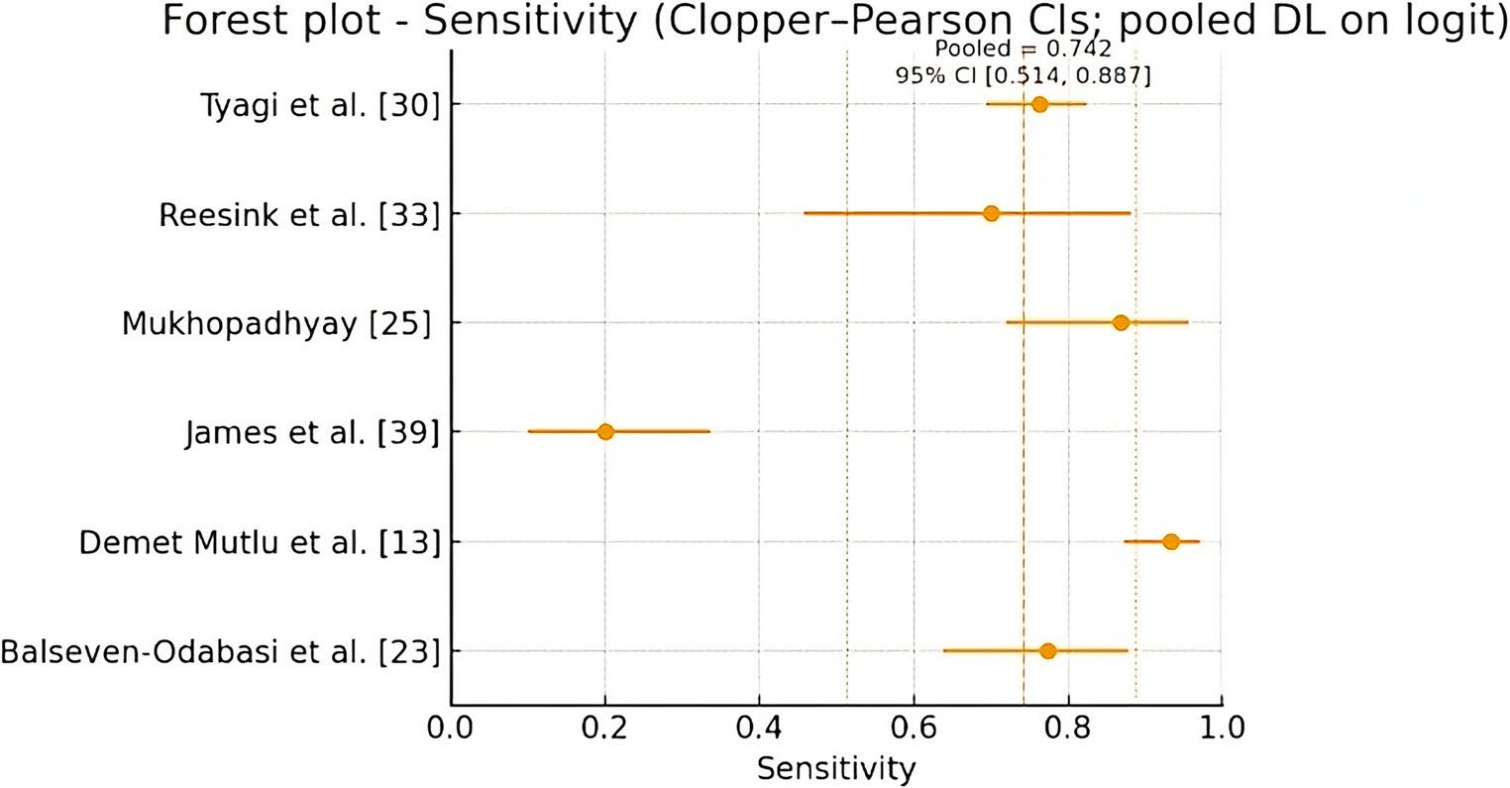
Forest plot for Sensitivity (*CI* confidence interval; *DL* DerSimonian–Laird method)

**Fig. 5:**
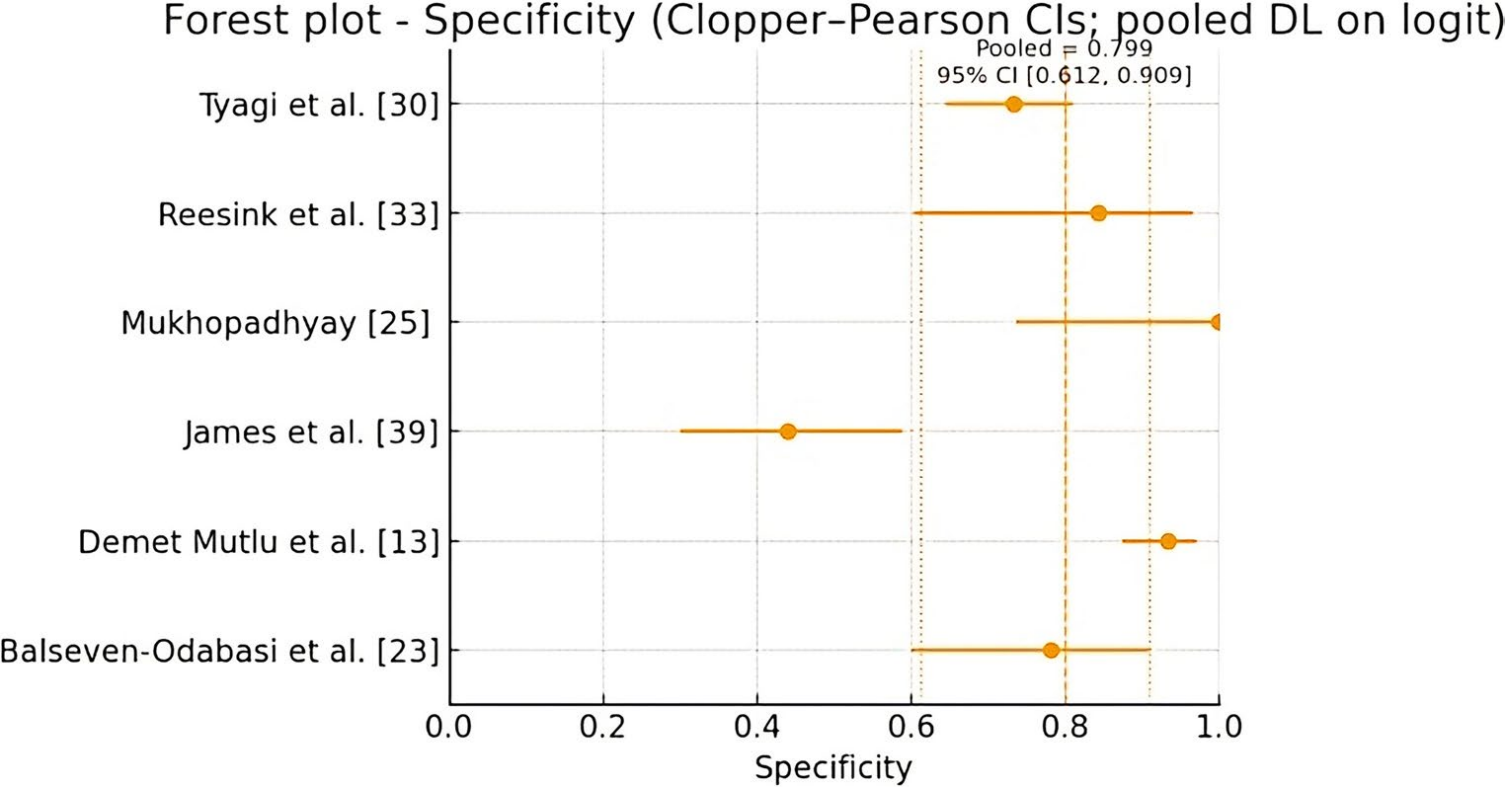
orest plot for Specificity (*CI* confidence interval; *DL* DerSimonian-Laird method)

Only 6 of the 23 studies included in the systematic review provided specific data for the meta-analysis, comprising a total of 827 participants. Figures 4 and 5 show the forest plots for sensitivity (Se) and specificity (Sp), respectively, with pooled values ​​of Se = 0.742 (95% CI) (0.514–0.887) and Sp = 0.799 (95% CI) (0.612–0.909). Regarding the assessment of heterogeneity, the statistics for each meta-analysis were: sensitivity I2 = 92.9% (considerable), Cochram’s Q = 70.3 and Tau2 = 1.43; and specificity I2 = 88.3% (considerable), Cochram’s Q = 42.83 and Tau2 = 1.04.

**Fig. 6:**
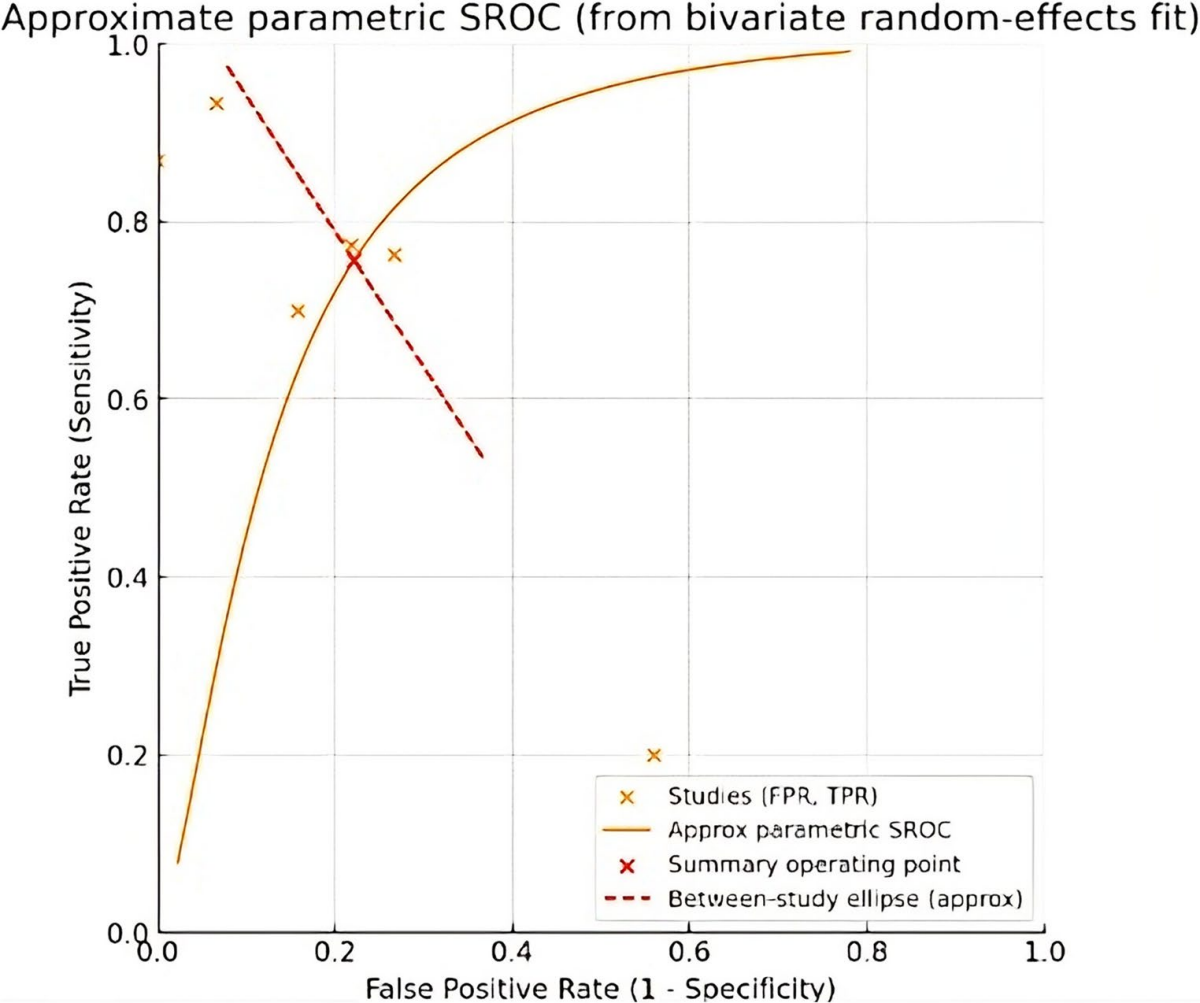
S-ROC curve parametric (*FPR* False Positive Rate, *TPR* True Positive Rate, *S-ROC* Summary Receiver Operating Characteristic curve)

Finally, based on the overall sensitivity and specificity results from the meta-analysis, the approximate parametric SROC curve was constructed (Fig. 6), where are shown the values of six studies included.

Taken together, the substantial proportion of unclear QUADAS-2 judgments, combined with heterogeneity in samples and analytic approaches, indicates that the certainty of the pooled sensitivity and specificity estimates should be regarded as low to moderate.

## Discussion

Among the 23 studies included in this systematic review, most reported statistically significant differences between males and females in at least one morphometric variable. Body dimensions of the hyoid bone (length, anteroposterior thickness, and transverse diameter) were the most frequently associated with sexual dimorphism. One of the most consistent findings is the superior discriminant capacity of body measurements compared to those of the greater horn, a trend observed across studies with diverse designs and geographical origins [23, 33, 34]. This difference may be explained by anatomical and developmental factors. The hyoid body provides multiple muscular attachments involved in swallowing and airway stabilization, and male hyoids tend to be larger overall, features that likely amplify dimorphism in body breadth and height [10]. In addition, the body ossifies early, whereas cornual fusion is later and often incomplete, increasing horn variability [9]. This contrast likely explains the superior discriminative performance of body measurements [11]. Taken together, these aspects help contextualize the observed differences across studies. Only one study examined bone density [9], but the reported accuracy was not notably higher than that obtained through metric variable analysis in the rest of the studies.

These classification outcomes must be viewed considering the methodological approaches adopted. A key observation is that multivariate discriminant analysis models improve sex classification, especially when combining variables from the hyoid body. This is supported by studies that reported accuracy levels above 75% using such statistical approaches [35, 40]. In contrast, other studies, with an overall accuracy less than 50%, highlight the variability in performance and the importance of rigorous design and appropriate variable selection [24].

Reported accuracy values vary widely, from values close to or even below random assignment, such as 44% in females [39] or 69.2% in males [36], to over 90% in those applying advanced analytical strategies [32, 41], particularly machine learning algorithms [13]. This heterogeneity appears closely linked to methodological factors, including sample size, population origin, fusion status, and statistical approach. Studies with stronger design reported higher accuracy rates [11, 30], sometimes reaching 83% when using complex models [34]. Our synthesis supports these findings, confirming the superior performance of multivariate over univariate methods [9, 33].

In quantitative terms, the present study also incorporated a meta-analysis of diagnostic accuracy. Pooled sensitivity (0.742; 95% CI: 0.514–0.887) and specificity (0.799; 95% CI: 0.612–0.909) indicate a moderate overall discriminatory capacity of the hyoid bone for sex estimation. These values fall within the broad range observed across individual studies. However, only six studies provided extractable 2 × 2 data, and substantial heterogeneity in populations, measurement techniques, and analytical approaches limits the generalizability of these pooled estimates. The wide confidence intervals further reflect underlying uncertainty, underscoring that the meta-analytic results should be interpreted cautiously.

In addition to these general trends, specific anatomical and demographic factors have also been shown to affect classification performance. Studies focusing on unfused or partially fused hyoids [25, 28, 29] obtained less consistent results, with greater overlap between sexes, particularly in younger samples or those with limited inclusion criteria. These findings emphasize that morphological variability of the hyoid may influence classification rates when factors such as age, lateralization, or ossification status are not adequately controlled.

Population origin also appears to influence the forensic applicability of the hyoid bone. Studies on samples from West Asia [23, 37], India [24, 25, 28–30, 39], and Central Europe [32] report marked differences in the means of the analyzed variables, underscoring the need to develop population-specific discriminant functions, especially for forensic or identification contexts. Likewise, the type of sample, whether dry bone or imaging-based, affects the morphometric values recorded, and consequently, model performance [13, 26, 38]. This supports the approach taken in this review, which considers the method of measurement (direct vs. indirect) as a relevant variable in the critical interpretation of findings [3]. With few exceptions [25, 34, 35], most studies using imaging techniques [3, 13, 23, 26, 37, 38] reported accuracy rates above 70%, outperforming those based on dry bone [24, 27, 39]. Finally, some recent studies implemented multivariate approaches, including logistic regression and ROC curve analysis, to refine sex classification models [27, 39]. While these studies demonstrate a moderate level of statistical complexity, they often lack transparency regarding variable selection or validation. Their limited accuracy (below 70%) likely reflects methodological issues such as small or unbalanced samples and absence of cross-validation.

Importantly, the overall certainty of the evidence should be regarded as low to moderate. This reflects the high proportion of unclear risk-of-bias judgments in the QUADAS-2 patient-selection (87%) and reference-standard domains (91%), together with limited methodological detail in many primary studies. These weaknesses reduce confidence in both the individual estimates and the pooled sensitivity and specificity and highlight the need for more transparent and methodologically robust research.

Regarding the meta-analysis revealed considerable heterogeneity in both sensitivity (I² = 92.9%) and specificity (I² = 88.3%), indicating marked variability among the included studies. This level of inconsistency suggests the presence of relevant differences in study design, populations analyzed, or diagnostic thresholds used. The magnitude of τ² supports the existence of real variation beyond chance, justifying the use of random-effects models. These findings highlight the need for caution in interpreting pooled estimates. They also underscore the importance of exploring clinical or methodological factors that may explain the observed heterogeneity.

Beyond these considerations, situating the performance of the hyoid bone within the broader forensic anthropology literature provides essential context. Pelvic morphology remains the most accurate skeletal indicator of sex, with revised Phenice-based methods achieving 94.5% cross-validated accuracy and 86.2% accuracy in independent validation samples [42]. Cranial trait analysis also yields consistently high performance, with discriminant-function approaches producing 88–90% correct classification [43]. In comparison, the pooled accuracy measures obtained in the present review, together with the low-to-moderate certainty of the evidence, indicate that the hyoid bone cannot match the discriminative reliability of the pelvis or skull. Accordingly, hyoid-based sex estimation should be used as a complementary tool, particularly when preservation is limited, rather than as a standalone method in forensic casework.

This review also highlights several limitations. One of the most recurrent methodological weaknesses is the absence of cross-validation or external validation procedures, which hinders the assessment of generalizability [25, 33, 36]. In over one-third of the studies, accuracy was reported only on the original sample, without replication testing. Variability in sample size and sex distribution further compromises model stability. Studies that used small or unbalanced samples [31, 35, 41]. In some cases, this issue is compounded by the absence of a clear rationale regarding the minimum sample size required to apply multivariate methods reliably. Regarding statistical analysis, inconsistency is also observed in how independent variables are selected and justified. Inconsistencies in variable selection and insufficient reporting of model-fit indices also raise concerns about transparency and replicability [3, 25, 31, 33, 36]. Population heterogeneity poses an additional challenge: while some studies clearly define the population origin [23, 34], others did not, limiting comparability and extrapolation. Differences in fusion status (fused, unfused, or transitional) adds structural variability that is not always addressed [27, 39].

## Conclusions

This systematic review suggests that the hyoid bone exhibits measurable sexual dimorphism and may be useful for sex estimation, particularly when traditional skeletal indicators are absent or compromised. Across the 23 studies included, the most consistent results were obtained through multivariate statistical models and machine learning techniques, with discriminant function analysis yielding accuracy rates between 69.2% and 93%, and artificial intelligence methods such as support vector machines and neural networks reaching up to 95.4%. Greater accuracy tended to occur in studies examining fused hyoids and in those with larger, more balanced samples. Variables related to the body of the hyoid, especially length and transverse dimensions, generally showed better discriminative performance than those of the greater horns. Moreover, classification accuracy tended to be slightly higher in females when advanced techniques were employed.

Regarding the meta-analysis, sensitivity and specificity were pooled across six studies, yielding a global diagnostic accuracy of 0.742 (95% CI: 0.514–0.887) and 0.799 (95% CI: 0.612–0.909), respectively. The width of these confidence intervals underscores the uncertainty of the pooled estimates, reflecting heterogeneity in study design, sample composition, and measurement approaches.

Population origin and age-related changes, particularly ossification status, appeared to influence morphometric patterns and classification performance. This highlights the importance of developing population-specific discriminant functions and accounting for variables such as body mass index, degree of fusion, and laterality. Imaging-based analysis tended to report slightly higher accuracy than dry-bone studies, suggesting a potential advantage of radiological methods in forensic applications.

However, the overall findings should be interpreted with caution due to considerable methodological heterogeneity, frequent unclear risk-of-bias judgements, and the limited external validation in several primary studies. Future research should prioritise broader and more representative population samples, systematic validation strategies, and standardised measurement protocols. Incorporating automated and rigorously validated machine-learning tools may further improve the robustness and reproducibility of sex estimation from the hyoid bone in both forensic and archaeological contexts.

### Limitations

This systematic review has several limitations. First, only studies published in English and Spanish and available in full text were included, which may have led to the exclusion of relevant data. Second, substantial variability in study design, population origin, sex distribution, and hyoid ossification status limited the comparability of primary outcomes and contributed to the heterogeneity observed in the synthesis. Third, inconsistency in statistical modelling, frequent absence of validation strategies, and insufficient reporting across several QUADAS-2 domains further constrain the interpretability and robustness of the available evidence. Additionally, although QUADAS-2 was applied to assess risk of bias, only six studies provided extractable 2 × 2 data, which restricted the scope of the meta-analysis. Finally, the STROBE checklist was used solely to evaluate reporting completeness. Applying a < 50% reporting-completeness threshold as an inclusion criterion ensured that sufficient methodological information was available for reliable data extraction and interpretation, but it may have introduced selection bias and reduced the representativeness of the included evidence.

## Supplementary Information

Below is the link to the electronic supplementary material.

Supplementary Material 1 (DOCX 61.1 KB) 
