# Supplementary material for: Is there sufficient evidence that the hyoid bone can be used for sexual dimorphism? a systematic review and meta-analysis
Source: Int J Legal Med. 2026 Feb 11;140(3):1513–28. doi: 10.1007/s00414-026-03722-3 (PMC13161326; doi:10.1007/s00414-026-03722-3)
Supplement: Supplementary file 1 — Supplementary Material 1 (DOCX 61.1 KB) [file 414_2026_3722_MOESM1_ESM.docx]

**Is there sufficient evidence that the hyoid bone can be used for sexual dimorphism? A systematic review and meta-analysis**

**Supplementary Table 1**. Databases and database-specific search strategies

| **Database** | **Search strategies** |
| --- | --- |
| PubMed  *Filter: Title, abstract* | 1. ((((((((((((((((((((sex characteristic) OR (sexual dimorphism)) OR (sex differences)) OR (sex dimorphism)) OR (sex determination by skeleton)) OR (determine sex)) OR (sex determination)) OR (determined sex)) OR (determining sex)) OR (estimate sex)) OR (sex estimation)) OR (estimated sex)) OR (estimating sex)) OR (sex assessment)) OR (assessed sex)) OR (assessing sex)) OR (sex-related)) OR (gender dimorphism)) OR (gender characteristic)) OR (gender difference)) 2. ((((hyoid bone) OR (throat bone)) OR (laryngohyoid complex)) OR (laryngohyoid structures)) 3. 1 AND 2 |
| Web of Science  *Filter: Topic* | 1. TS=(((((((((((((((((((((sex characteristic) OR (sexual dimorphism)) OR (sex differences)) OR (sex dimorphism)) OR (sex determination by skeleton)) OR (determine sex)) OR (sex determination)) OR (determined sex)) OR (determining sex)) OR (estimate sex)) OR (sex estimation)) OR (estimated sex)) OR (estimating sex)) OR (sex assessment)) OR (assessed sex)) OR (assessing sex)) OR (sex-related)) OR (gender dimorphism)) OR (gender characteristic)) OR (gender difference)) 2. TS=(((((hyoid bone) OR (throat bone)) OR (laryngohyoid complex)) OR (laryngohyoid structures))) 3. 1 AND 2 |
| Scopus  *Filter: Title, abstract, keywords* | 1. ( TITLE-ABS-KEY ( ( ( ( ( ( ( ( ( ( ( ( ( ( ( ( ( ( ( ( ( sex AND characteristic )   OR ( sexual AND dimorphism ) ) OR ( sex AND differences ) ) OR ( sex AND dimorphism ) ) OR ( sex AND determination AND by AND skeleton ) ) OR ( determine AND sex ) ) OR ( sex AND determination ) ) OR ( determined AND sex ) ) OR ( determining AND sex ) ) OR ( estimate AND sex ) ) OR ( sex AND estimation ) ) OR ( estimated AND sex ) ) OR ( estimating AND sex ) ) OR ( sex AND assessment ) ) OR ( assessed AND sex ) ) OR ( assessing AND sex ) ) OR ( sex-related ) ) OR ( gender AND dimorphism ) ) OR ( gender AND characteristic ) ) OR ( gender AND difference ) ) )   1. TITLE-ABS-KEY ( ( ( ( ( hyoid AND bone ) OR ( throat AND bone ) ) OR ( laryngohyoid AND complex ) ) OR ( laryngohyoid AND structures ) ) ) ) 2. 1 AND 2 |

**Supplementary Table 2**. Morphometric variables and main conclusions

| **Author** | **Method** | **Variables** | **Conclusion** |
| --- | --- | --- | --- |
| Balseven-Odabasi et al. [23] | Metric analysis (33 variables). | Relevant for DFA: 1. Distance between greater horn tips 2. Perpendicular distance from horn tips to center of body 3. Max length of left lesser horn | Population-specific differences were observed. Full-variable models showed better performance than reduced ones. |
| Demet Mutlu et al. [13] | Metric analysis (8 variables). | 1. Body length (BL) 2. Body height (BH) 3. Right greater horn length (RCL) 4. Left greater horn length (LCL) 5. Internal length (HIL) 6. External length (HEL) 7. Internal width (HIW) 8. External width (HEW) | High accuracy was achieved. Dimorphism varied by population |
| Fakhry et al. [3] | Metric analysis + morphological analysis (12 variables) | 1. Distance A (width between greater horns) 2. Distance B (length from body to horn tips) 3. Distance C (body width between lesser horns) 4. Alpha angle (between greater horns) | Hyoid features significantly varied by sex, stature, and weight. Sexual dimorphism was marked and influenced by anatomical (length, width and horn angles) and functional factors (muscle traction in living individuals). |
| Fisher et al. [9] | Fusion grade analysis + bone density analysis | Fusion types: 1. Unilateral/partial 2. Bilateral 3. No bilateral fusion 4. Distant non-fusion Bone density (Hounsfield units) | Bone density and fusion status aided sex and age estimation, especially in females (lower in adult females and decreased with age). |
| Guliani et al. [24] | Metric analysis (6 variables) | 1. Total external length (LET) 2. Distance between horns (DA) 3. Horn length (LA) 4. Anteroposterior length (AP) 5. Body height (HB) 6. Body width (WB) | External length was the best predictor. Males had larger hyoids. |
| Haj Salem et al. [37] | Metric analysis (10 variables). | 1. Distal length of greater cornua (center). 2. Distal length of greater cornua (parietal) 3. Max. length of right greater cornu 4. Max. length of left greater cornu  5. Body width (joint space to joint space)  6. Perpendicular from cornua line to anterior body  7. Perpendicular from cornua line to posterior body  8. Mid-body width (perpendicular)  9. Distal diameter of right greater cornu  10. Distal diameter of left greater cornu | Several variables showed high significance (M3, M4, M5, M6, M7). Final model based on two (M4 and M6) yielded >70% accuracy. |
| James et al. [39] | Metric analysis (7 variables). | 1. Total length (THL) 2. Total width (THW) 3. Body height (BH) 4. Body length (BL) 5. Horn height (CHI) 6. Horn length (CL) 7. Body thickness (BT) | Total and body length were significant predictors of sex. Low Kappa score indicated weak predictive reliability. |
| Kindschuh et al. [34] | Metric analysis (10 variables) | 1. Maximum body length (BL) 2. Maximum body height (BH) 3. Horn width at fusion (inferior end) (CWI) 4. Horn height at fusion (inferior end) (CHI) 5. Maximum length of the greater horn (CL) 6. Maximum width of horn (superior end) (CWS) 7. Maximum height of horn (superior end) (CHS) 8. Total length (THL) 9. Total width (THW) 10. Total width between horn tips (superior ends) (WCS) | Sexual dimorphism confirmed even in unfused hyoids. Body alone allowed accurate sex estimation. |
| Köse et al. [40] | Metric analysis (8 variables) | 1. Width (Hyd-W) 2. Body length (B-L) 3. Hyoid length (Hyd-L) 4. Left proximal width (LP-W) 5. Right proximal width (RP-W) 6. Left distal diameter (LD-D) 7. Right distal diameter (RD-D) 8. Total bilateral length | Cone beam CT was effective. Body length and bilateral length had highest sex estimation accuracy (>80%) |
| Logar et al. [35] | Metric analysis (15 variables). | Most relevant: 1. Maximum body length (BL) 2. Maximum body height (BH) 3. Articular facet width at body-horn junction (CWI) 4. Articular facet height at body-horn junction (CHI) 5. Maximum greater horn length (CL) 6. Maximum superior horn width (CWS) (Fused only): 7. Maximum superior horn height (CHS) 8. Total length (THL) 9. Total width (THW) | Significant sexual dimorphism observed. Univariate DFA yielded >90% accuracy. |
| Miller et al. [36] | Metric analysis (31 variables) | Most relevant  1. Distances from horn tip to body 2. Articular space width 3. Distal horn tip distances 4. Midline-perpendicular distance to posterior body | Most variables were larger in males. Length-related measures showed stronger dimorphism than width. |
| Mukhopadhyay [25] | Metric analysis (6 variables) | 1. Body width (BC)  2. AP length (AP) 3. Horn length (CR)  4. Body width (AW) 5. Lesser horn base distance (DR)  6. Slope (S) | 90% sex classification accuracy in Bengali population. Male hyoids were generally larger. |
| Naimo et al. [38] | Metric analysis (17 variables). | Most relevant:  1. Inter-horn tip distance 2. Horn angle (L2, L3) 3. Mid-body length 4. Distal horn length (L/R) 5. Horn base distances 6. Horn sagittal angles (L/R) | Sexual differences emerged during adolescence. No significant differences in horn shape between sexes. |
| Okasi et al. [26] | Metric analysis (9 variables) | Most relevant:  1. Distance horn-body 2. Body diameter, Body length 3. Vertical horn length 4. Horn angles and inter-horn angles | Male hyoids were generally larger. Left greater horn length was the best predictor. |
| Pollard et al. [31] | Metric analysis (6 variables) | 1. Width between horn tips 2. Length from body to horn tips 3. Lesser horn spacing 4. Left horn end spacing  5. Right horn end spacing 6. Alpha angle | Hyoid shape related to sex and build. Findings informed fracture mechanism in strangulation cases |
| Reesink et al. [33] | Metric analysis (13 variables) | Most relevant: 1. Maximum medial body height (MMH) 2. Anteroposterior body thickness (ATP) 3. Body curvature depth (DPS) 4. Combined greater horn length (CML) 5. Maximum transverse body diameter (MTD) 6. Maximum distance between body and horn 7. Lesser horn length 8. Distance between bases of lesser horns 9. Distance between greater horn tips 10. Length of greater horn tips | Single variable insufficient; combining three improved classifications (MMH, ATP and MTD); only one showed clear significance (MTD). |
| Soltani et al. [11] | Metric analysis (3 variables) | 1. Distance A (greater horn width) 2. Distance B (lesser horn width) 3. Distance C (hyoid length) | Hyoid length and minor horn distance were sex predictors. Length was most relevant. |
| Soltani et al. [27] | Metric analysis (4 variables) | 1. Horn width  2. Body AP diameter 3. Body height  4. Posterior concavity depth | Four measures showed sex differences, and all were larger in males. |
| Torimitsu et al. [41] | Metric analysis (7 variables) | 1. Width (HW) 2. Body length (BL) 3. Hyoid length (HL) 4. Left proximal width (LPW) 5. Right proximal width (RPW) 6. Left distal diameter (LDD) 7. Right distal diameter (RDD) | Strong dimorphism in Japanese population but not generalizable. CT useful for analysis. |
| Tyagi et al. [28] | Weight analysis (4 variables) | 1. Weight of right non-fused greater horn (WRC) 2. Weight of left non-fused greater horn (WLC) 3. Weight of non-fused body (WB) 4. Total non-fused hyoid weight (WCH) | Hyoid weight was greater in males. DFA yielded accuracy rates of up to 76.6% in females. Weight alone was insufficient; combining variables was recommended. |
| Tyagi et al. [29] | Weight analysis (3 variables) | 1. Weight of fused part (WFP) 2. Weight of unfused part (WUP) 3. Total bone weight (WCH) | Fusion status affected weights. DFA accuracy ranged 71.7–75.5%, higher in females. Weight alone was insufficient; combining with other morphometric data was necessary. |
| Tyagi et al. [30] | Metric analysis (12 variables). | Most relevant: 1. Body length (BL) 2. Body height (BH) 3. Right/left greater horn length (RCL/LCL) 4. Horn width at joint (right/left) (RCWA/LCWA) 5. Horn height at joint (right/left) (RCHA/LCHA)  6. Right/left distal horn width (RCWD/LCWD) 7. Right/left distal horn height (RCHD/LCHD) | Multivariate DFA was effective. Small sample limited robustness. Machine learning methods were encouraged. |
| Urbanová et al. [32] | Metric analysis (9 variables) + morphological analysis (23 variables) | 1. Total length (THL) 2. Total width (THW) 3. Body height (BH) 4. Body length (BL) 5. Greater horn height at anterior end (CHIdx) 6. Greater horn height at fusion line (CHIsin) 7. Greater horn length at central point (CLdx) 8. Greater horn length at distal end (CLsin) 9. Superior anteroposterior body thickness (BT) | Symbolic regression outperformed DFA. Age affected accuracy in women. Linear metrics were more effective than shape. |
| DFA: discriminant function analysis | | | |

**Supplementary Table 3**. Reporting completeness assessment of the 26 included studies based on the STROBE checklist for cross-sectional studies

| **Study ID** | **1a** | **1b** | **2** | **3** | **4** | **5** | **6** | **7** | **8** | **9** | **10** | **11** | **12a** | **12b** | **12c** | **12d** | **12 e** | **13a** | **13b** | **13c** | **Reporting-completeness Index** |
| --- | --- | --- | --- | --- | --- | --- | --- | --- | --- | --- | --- | --- | --- | --- | --- | --- | --- | --- | --- | --- | --- |
| [23] | No | Yes | Yes | Yes | No | Yes | Yes | No | Yes | No | No | Yes | No | Yes | NA | Yes | NA | Yes | NA | NA | 16/26= 61.50% |
| [13] | No | Yes | Yes | Yes | No | Yes | Yes | No | Yes | No | No | Yes | No | Yes | NA | NA | Yes | Yes | NA | NA | 14/26= 53.80% |
| [3] | No | Yes | Yes | Yes | No | Yes | Yes | No | Yes | No | No | Yes | No | Yes | NA | NA | Yes | Yes | NA | NA | 14/26= 53.80% |
| [9] | No | Yes | Yes | Yes | No | Yes | Yes | No | Yes | Yes | Yes | Yes | Yes | Yes | Yes | NA | NA | Yes | NA | NA | 21/27= 77.77% |
| [24] | Yes | Yes | Yes | No | Yes | Yes | Yes | No | Yes | No | No | Yes | No | Yes | NA | NA | NA | Yes | NA | NA | 14/25= 56% |
| [19] | No | Yes | Yes | No | No | Yes | Yes | No | Yes | No | No | Yes | No | Yes | NA | NA | NA | Yes | NA | NA | 12/25= 48% |
| [8] | No | Yes | Yes | No | No | Yes | Yes | No | Yes | No | No | Yes | No | Yes | NA | NA | NA | Yes | NA | NA | 11/25= 44% |
| [37] | Yes | Yes | Yes | Yes | Yes | Yes | Yes | No | Yes | No | No | Yes | No | Yes | NA | Yes | Yes | Yes | NA | NA | 18/27= 66.60% |
| [39] | No | Yes | Yes | Yes | No | Yes | Yes | No | Yes | No | Yes | Yes | No | Yes | NA | NA | NA | Yes | NA | NA | 15/25= 60% |
| [20] | No | Yes | Yes | No | No | Yes | Yes | No | Yes | No | No | Yes | No | Yes | NA | NA | Yes | Yes | NA | NA | 11/26= 42.30% |
| [34] | No | Yes | Yes | No | No | Yes | Yes | Yes | Yes | Yes | No | Yes | Yes | Yes | NA | NA | Yes | Yes | NA | NA | 19/26= 73.07% |
| [40] | No | Yes | Yes | Yes | No | Yes | Yes | No | Yes | No | No | Yes | No | Yes | NA | NA | Yes | Yes | NA | NA | 17/26=65.38% |
| [35] | No | Yes | Yes | No | No | Yes | Yes | No | Yes | Yes | No | Yes | No | Yes | NA | NA | Yes | Yes | NA | NA | 19/26= 73.07% |
| [36] | No | Yes | Yes | No | No | Yes | No | No | Yes | Yes | No | Yes | No | Yes | NA | NA | Yes | Yes | NA | NA | 18/27= 66.60% |
| [25] | No | Yes | Yes | No | No | Yes | Yes | No | Yes | No | No | Yes | No | Yes | NA | NA | NA | Yes | NA | NA | 14/25= 56% |
| [38] | No | Yes | Yes | Yes | No | Yes | No | No | Yes | No | No | Yes | No | Yes | NA | NA | NA | Yes | NA | NA | 13/25= 52% |
| [26] | No | Yes | Yes | No | No | Yes | Yes | No | Yes | No | Yes | Yes | No | Yes | NA | NA | NA | Yes | NA | NA | 16/25= 64% |
| [31] | No | Yes | Yes | Yes | No | Yes | Yes | Yes | Yes | Yes | No | Yes | No | Yes | NA | NA | NA | Yes | NA | NA | 19/25= 76% |
| [33] | No | Yes | Yes | No | No | Yes | No | No | Yes | Yes | No | Yes | No | Yes | NA | NA | NA | Yes | NA | NA | 13/25= 52% |
| [11] | Yes | Yes | Yes | Yes | Yes | Yes | Yes | Yes | Yes | Yes | No | Yes | Yes | Yes | NA | Yes | NA | Yes | NA | NA | 23/26= 88.46% |
| [27] | No | Yes | Yes | Yes | No | Yes | Yes | No | Yes | No | No | Yes | Yes | Yes | NA | NA | NA | Yes | NA | NA | 16/25= 64% |
| [41] | No | Yes | Yes | No | No | Yes | Yes | No | Yes | Yes | No | Yes | No | Yes | NA | NA | Yes | Yes | NA | NA | 17/26= 65.38% |
| [28] | No | Yes | Yes | Yes | No | Yes | Yes | No | Yes | No | No | Yes | No | Yes | NA | NA | NA | Yes | NA | NA | 15/25= 60% |
| [29] | No | Yes | Yes | No | No | Yes | Yes | No | Yes | No | No | Yes | No | Yes | NA | NA | Yes | Yes | NA | NA | 13/26= 50% |
| [30] | No | Yes | Yes | Yes | No | Yes | Yes | No | Yes | No | No | Yes | No | Yes | NA | NA | Yes | Yes | NA | NA | 15/26= 57.70% |
| [32] | No | Yes | Yes | Yes | No | No | Yes | No | Yes | No | No | Yes | No | Yes | NA | NA | Yes | Yes | NA | NA | 15/26= 57.70% |
| Note: STROBE item numbers correspond to the official checklist for cross-sectional studies (www.strobe-statement.org). Abbreviation: NA = Not applicable. | | | | | | | | | | | | | | | | | | | | | |

| **Study ID** | **14a** | **14b** | **15** | **16a** | **16b** | **16c** | **17** | **18** | **19** | **20** | **21** | **22** |
| --- | --- | --- | --- | --- | --- | --- | --- | --- | --- | --- | --- | --- |
| [23] | No | NA | Yes | No | No | NA | No | Yes | Yes | Yes | Yes | Yes |
| [13] | No | NA | Yes | No | No | NA | No | Yes | No | Yes | No | Yes |
| [3] | No | NA | Yes | No | No | NA | No | Yes | No | Yes | No | Yes |
| [9] | Yes | Yes | Yes | No | No | NA | Yes | Yes | Yes | Yes | No | Yes |
| [24] | No | NA | Yes | No | No | NA | No | Yes | No | Yes | No | Yes |
| [19] | No | NA | Yes | No | No | NA | No | Yes | No | Yes | No | Yes |
| [8] | No | NA | Yes | No | No | NA | No | Yes | No | Yes | No | No |
| [37] | No | NA | Yes | Yes | No | NA | No | Yes | Yes | No | Yes | No |
| [39] | No | NA | Yes | No | No | NA | No | Yes | Yes | Yes | Yes | No |
| [20] | No | NA | Yes | No | No | NA | No | Yes | No | No | No | No |
| [34] | No | NA | Yes | No | No | NA | Yes | Yes | Yes | Yes | Yes | Yes |
| [40] | No | NA | Yes | Yes | Yes | NA | Yes | Yes | No | Yes | Yes | No |
| [35] | Yes | NA | Yes | Yes | Yes | NA | Yes | Yes | No | Yes | Yes | Yes |
| [36] | Yes | NA | Yes | Yes | Yes | NA | Yes | Yes | No | Yes | Yes | Yes |
| [25] | Yes | NA | Yes | Yes | Yes | NA | No | Yes | No | No | No | Yes |
| [38] | Yes | NA | Yes | No | Yes | NA | No | Yes | Yes | No | No | No |
| [26] | Yes | NA | Yes | Yes | No | NA | No | Yes | Yes | Yes | No | Yes |
| [31] | Yes | NA | Yes | No | Yes | NA | Yes | Yes | No | Yes | Yes | Yes |
| [33] | No | NA | Yes | No | No | NA | No | Yes | No | Yes | Yes | Yes |
| [11] | Yes | NA | Yes | Yes | Yes | NA | No | Yes | Yes | Yes | Yes | No |
| [27] | Yes | NA | Yes | Yes | Yes | NA | No | Yes | No | No | Yes | No |
| [41] | Yes | NA | Yes | No | Yes | NA | Yes | Yes | No | Yes | Yes | No |
| [28] | No | NA | Yes | No | No | NA | No | Yes | Yes | Yes | Yes | Yes |
| [29] | No | NA | Yes | No | Yes | NA | No | Yes | No | Yes | No | No |
| [30] | No | NA | Yes | No | No | NA | No | Yes | No | Yes | Yes | Yes |
| [32] | No | NA | Yes | No | No | NA | No | Yes | Yes | Yes | Yes | Yes |
